# Nutrient optimization in bioleaching: are we overdosing?

**DOI:** 10.3389/fmicb.2024.1359991

**Published:** 2024-05-17

**Authors:** Carmen Falagán, Tomasa Sbaffi, Gwion B. Williams, Rafael Bargiela, David W. Dew, Karen A. Hudson-Edwards

**Affiliations:** ^1^Environment & Sustainability Institute and Camborne School of Mines, University of Exeter, Cornwall, United Kingdom; ^2^Molecular Ecology Group, Water Research Institute (IRSA), National Research Council of Italy (CNR), Rome, Italy; ^3^National Biodiversity Future Centre, NBFC, Palermo, Italy; ^4^Centre for Environmental Biotechnology (CEB), School of Natural Sciences, Bangor University, Bangor, United Kingdom

**Keywords:** bioleaching, nitrogen, ammonium, phosphorous, phosphate, nutrients, microbial community, media composition

## Abstract

The general trend in biomining (i.e., bioleaching and biooxidation) is the use of media with high concentrations of the nutrients (nitrogen as ammonium, phosphorous as phosphate, and K), which are considered to be essential for microbial growth. The depletion of any of the nutrients would affect negatively the bioleaching (and biooxidation) capacity of the microorganisms, so the formulation of the different media ensures that there is a surplus of nutrients. However, some of these nutrients (e.g., phosphate, K) may be already present in the ore and are made available to the microorganisms when the ore is exposed to the low-pH media used during bioleaching. The effect of phosphate addition (109 mg/L) and depletion on the bioleaching of low-grade sulfidic ore alongside the determination of ammonium (i.e., 25 mg/L, 50 mg/L, 109 mg/L, 409 mg/L, and 874 g/L) requirements were studied. The results of the experiments presented showed that the addition of phosphate did not have any effect on the bioleaching of the low-grade sulfidic ore while the addition of ammonium was necessary to obtain higher redox potentials (>650 mV vs. Ag/AgCl) and higher metal (Co, Cu, Ni, and Zn) dissolutions. Temperature was the factor that shaped the microbial communities, at 30°C, the microbial community at the end of all the experiments was dominated by *Acidithiobacillus* sp. as well as at 42°C, except when nutrients were not added and *Sulfobacillus* sp. was the dominant microorganism. At 55°C, DNA recovery was unsuccessful, and at 60°C, the microbial communities were dominated by *Sulfolobus* sp. In conclusion, the amount of nutrients in bioleaching could be reduced significantly to achieve the redox potentials and metal dissolution desired in bioleaching without affecting the microbial communities and bioleaching efficiencies.

## 1 Introduction

Bioleaching aims at extracting metals (e.g., Co, Cu, Ni, and Zn) from ores and wastes (e.g., tailings, waste rock, and printed circuit boards) using acidophilic microorganisms (Johnson et al., 2023). Cultivation of acidophiles and bioleaching process optimization are particularly important to optimize metal extraction rates. The target microorganisms in bioleaching are chemolithoautotrophic acidophiles (e.g., *Acidithiobacillus* spp., *Leptospirillum* spp.) which use atmospheric CO_2_ as a carbon source and Fe or S as an energy source. Acidophiles are frequently grown in media with nutrients and/or trace elements. This is particularly important when the microbes are cultured in the absence of ore which can provide the necessary macro- and micronutrients for their continuous growth. Hallberg and Johnson (2007) developed a medium for the growth and isolation of acidophiles that contains basal salts and trace elements. This media contains the nutrients ammonium (41 mg/L), phosphate (35 mg/L) and K (29 mg/L), (see Ňancucheo et al., 2016 for full media composition). Other media used also for the isolation of acidophiles have similar chemical compositions but do not contain trace elements and have different amounts of nutrients (55–109 mg/L ammonium, 27–109 mg/L phosphate, 13–45 mg/L K); (Norris et al., 1996, 2020; Coram and Rawlings, 2002).

Higher concentrations of ammonium, phosphate and K are used in bioleaching experiments with chemolithoautotrophic acidophiles compared to those used in the growth and isolation of acidophiles. The medium 9 K medium, developed in 1959 by Silverman and Lundgren (1959) and originally developed for the cultivation of *Acidithiobacillus ferooxidans*, is widely used in bioleaching. This medium contains 49 mg/L Mg, 819 mg/L ammonium, 273 mg/L phosphate and 165 mg/L K. Hoffmann et al. (1981) showed that iron oxidation by *A. ferrooxidans* in a low-phosphate/low-sulfate medium without ammonium (102 mg/L Mg, 0 mg/L ammonium, 70 mg/L phosphate and 75 mg/L K) was more efficient than in 9 K medium due the high concentration of phosphate causing the formation of insoluble Fe-phosphates salts. However, most media used in batch mode bioleaching experiments, either in shake flasks or in reactors, are based on the 9 K medium, with additions of 4–59 mg/L Mg, 82–1,010 mg/L ammonium, 27–1908 mg/L phosphate and 22–786 mg/L K (Chen and Lin, 2001; Rodrıguez et al., 2003; Vilcáez et al., 2008; Zhou et al., 2009; Akinci and Guven, 2011; Spolaore et al., 2011; Lotfalian et al., 2015; Hubau et al., 2020). Some authors have added nutrient trace elements (e.g., Zn, Cu, Co, and Mn) to the media (Falagán et al., 2017; Santos et al., 2020), but this may be unnecessary if these are present in the ore minerals.

Laboratory scale-columns are used to study heap bioleaching at laboratory scale to understand the factors controlling bioleaching performance before and during mine operation. The media used in such experiments normally contain lower amounts of nutrients than media used in batch-mode bioleaching experiments. The columns are filled with crushed ore minerals and irrigated in continuous mode with an acidic solution which provides a continuous input of nutrients (e.g., Norris et al., 2012a,b; Manafi et al., 2013). For example, Norris et al. (2012a,b) used a medium containing 4 mg/L Mg, 5 mg/L ammonium, 5 mg/L phosphate and 2 mg/L K for the bioleaching of a copper sulfide ore and of a polymetallic sulfide ore using lab-scale columns. Manafi et al. (2013) compared the performances of the 9 K medium and a medium containing 819 mg/L ammonium, 485 mg/L phosphate and 315 mg/L K on the bioleaching of a pyritic porphyry copper sulfide ore in laboratory-scale columns. At the end of the experiments, all the columns (except the control column irrigated with biocide agent) had similar redox values and high Cu extraction yields (90–91%) that did not vary in the presence or absence of phosphate.

These studies show that the general trend in bioleaching is adding high concentration of nutrients (e.g., 9 K medium). However, some of the nutrients are likely present in the ore (e.g., K, Mg, and phosphate) and thus, their addition may be unnecessary. To date, there are no studies that focus on media optimization used in bioleaching. Some batch-mode bioleaching experiments and continuous mode laboratory-scale columns (e.g., Chen and Lin, 2001; Rodrıguez et al., 2003; Zhou et al., 2009; Norris et al., 2012a,b) have shown that low concentration of nutrients does not affect the bioleaching effectiveness. These highlights the necessity of studying the effect of addition/depletion of nutrients on the microbial communities and on the bioleaching process. This is significant in bioleaching at industrial scale as nutrient addition may increases cost of operation and particularly in bioleaching of low-grade ores and mine wastes (e.g., tailings). The low metal content of these resources makes them unsuitable for metal extraction. Therefore, the present study aims to investigate the effect of addition or depletion of nutrients on bioleaching efficiency during the bioleaching a low-grade ore. Metal extraction rates, Fe-oxidation, and microbial community compositions under varying nutrient conditions, specifically focusing on ammonium and phosphate, were assessed.

## 2 Low-grade ore background

The low-grade ore used in the present study was provided by the Sotkamo Mine in Finland (Terrafame Mining Company) which is a heap bioleaching operation that produces Ni, Zn and Co from pyrrhotite-rich pentlandite ore. The ore is bioleached in two stages; in a primary heap leach where remains for approximately 18 months and then in a secondary heap (the ore then is referred to as secondary ore) where leaching continues for at least 3 years (Arpalahti and Lundström, 2018). The heap pads are continuously irrigated with a low pH solution (~ pH 2.0) that is not amended with nutrients (e.g., ammonium and phosphate) but contains high concentration of metal(oid)s (Fe, Mn, Zn, Al, Ni, Mg) (Arpalahti and Lundström, 2019).

Research using ore and solutions from the Sotkamo Mine had been carried out for several decades. Niemelä et al. (1994) assessed the effect of ammonium, nitrate and phosphate addition on the bioleaching of the Sotkamo ore (the study did not specify if the ore used was primary or secondary ore). The experiments were carried out using shake flasks at 30°C and at 35°C and at pH 1.5 with 5 (wt./v) of ore. The authors determined that while nitrate was toxic, amendment with ammonium enhanced microbial Fe oxidation and phosphate had no effect. Ahoranta et al. (2017) used process liqueurs from the Sotkamo Mine to show that ammonium addition increased indigenous microbial Fe oxidation but phosphate, K and Na had no effect. Hubau et al. (2020) used 2-L batch reactors to bioleach the secondary ore (10% wt./v pulp density) at 42°C, 48°C, and 55°C using three different microbial consortia. The 42°C consortium was constructed from isolates kept at the French Geological Survey (BRGM) and was dominated by *Leptospirillum*, *Acidithiobacillus* and *Sulfobacillus*. The consortium used for the experiments at 48°C and 55°C was obtained at the University of Exeter (Fonti et al., 2019). The 48°C was dominated by *Acidithiobacillus*, *Acidithiomicrobium*, and *Sulfobacillus* while the 55°C was the same consortium than the one used at 48°C but it was cultivated, before inoculation of the reactors, with the Sotkamo secondary ore. The authors used the 0Km medium that contained 51 mg/L Mg, 1,009 mg/L ammonium, 667 mg/L phosphate, and 335 mg/L K. Their results showed that dissolution of Co clearly improved by the microbial activity, Cu dissolution was higher at 55°C than at 42°C and 48°C, while Ni was rapidly dissolved suggesting that it was contained in ready-soluble salts and its dissolution was not mediated by microbial activity. Hubau et al. (2024) determined the effect of nutrient concentration (1,009 mg/L and 101 mg/L ammonium, 334 and 67 mg/L K) on the bioleaching of the same secondary ore with 2-L batch reactors and at 20% wt./v of pulp density. The authors used a consortium dominated by candidate genus “*Fervidacidithiobacillus*” (formerly *Acidithiobacillus*), “*Acidithiomicrobium*”, and *Sulfobacillus*. Their results showed that the lower ammonium concentration had a negative impact on the microbial iron oxidizing activity and microbial growth while the effect of lower amounts of K was more noticeable on the microorganisms during the stationary phase but had no impact on achieving high redox potential. Hubau et al. (2024) showed that the metal dissolution achieved was similar in all the experiments independent of the nutrient concentration used. Falagán et al. (2024) studied the effect of inoculation and nutrient addition and Al and Mg in irrigation solution on metal extraction rates from the secondary ore at 48°C and at 60°C. The authors determined that inoculation and irrigation with nutrient solution (49 mg/L ammonium, 49 mg/L phosphate, and 40 mg/L K) favored microbial Fe oxidation and metal dissolution of the secondary ore compared to the experiments that were not inoculated and irrigated with nutrient-free solution.

The studies carried out with the secondary ore highlight the importance of nutrient addition during bioleaching, the effect of ammonium seeming particularly important. However, the effect of varying nutrient concentrations on the indigenous microbial communities is unknown. The present study investigates the bioleaching efficiency of indigenous microbial communities, their responses under decreasing concentrations of ammonium and depletion of phosphate, and provides a medium formulation for the bioleaching of the secondary ore and other similar low-grade ores including tailings and other mine wastes.

## 3 Materials and methods

### 3.1 Mineral

The low-grade ore used in the present study was the secondary ore obtained from the Sotkamo mine (Terrafame Mining Company, Finland). The main minerals present in the secondary ore are quartz (24.7 wt. %), K-feldspar (14.8 wt. %), jarosite (11.2 wt. %), any other Fe secondary minerals with Fe, S and O (12.0 wt. %), plagioclase (8.3 wt. %), phlogopite (5.8 wt. %), tremolite (4.0 wt. %), chlorite (2.7 wt. %), gypsum (2.7 wt. %), Fe-oxides (27 wt. %), pyrite (2.5 wt. %) muscovite (2.3 wt. %), magnesiochlorite (1.8 wt. %). For the experiments described in this paper, samples of secondary ore were milled to <300 μm diameter. Metal concentrations in the sample of secondary ore used in this study were 0.01 wt. % Co, 0.14 wt. % Cu, 6.84 wt. % Fe, 0.12 wt. % Ni and 0.32 wt. % Zn.

### 3.2 Microbial enrichments

Unmilled dried secondary ore and pregnant leaching solution were sampled from the secondary ore in sterile conditions and used to obtain microbial enrichments. The enrichment cultures were grown in media containing trace elements (as 10 mg/L ZnSO_4_·7H_2_0, 1 mg/L CuSO_4_·5H_2_O mg/L, 1 mg/L MnSO_4_·4H_2_O, 1 mg/L CoSO_4_·7H_2_O, 0.5 mg/L Cr_2_(SO_4_)_3_·15H_2_O, 0.6 mg/L H_3_BO_3_, 0.5 mg/L NaMoO_4_·2H_2_O, 1 mg/L NiSO_4_·6H_2_O, 1 mg/L Na_2_SeO_4_·10H_2_O, 0.1 mg/L Na_2_WO_4_·2H2O, and 0.1 mg/LNaVO_3_) and basal salts (as 150 mg/L (NH_4_)_2_SO_4_, 150 mg/L Na_2_SO_4_∙10H_2_O, 50 mg/L KCl, 500 mg/L MgSO_4_·7H_2_O, 50 mg/L KH_2_PO_4_, 14 mg/L Ca(NO_3_)_2_·4H_2_O) (Ňancucheo et al., 2016) and ferrous iron (1.4 g/L) or elemental sulfur (~1% wt./v) as energy sources and pH 1.8. Cultures were incubated at five different temperatures, 30°C, 42°C, 48°C, 55°C, and 60°C in order to obtain mesophilic, moderate thermophilic and thermophilic strains that could potentially be used in the heaps at the Sotkamo Mine. The enrichments did not contain any source of organic carbon so autotrophic microorganisms (i.e., use CO_2_ as a carbon source) would grow preferentially.

The growth of microorganisms in the enrichments was assessed by optical microscopy to observe proliferation of microbial cells, and by measuring redox potential. Microbial activity has been shown to increase the rate of Fe oxidation by at least five orders of magnitude at low pH conditions (Singer and Stumm, 1970; Nordstrom et al., 2015). Redox potential is used to assess iron speciation; ferrous iron is the dominant species when redox potential is low (< ~450 mV vs. Ag/AgCl) and ferric iron is the dominant species when redox potential is higher than 450 mV (*vs* Ag/AgCl).

### 3.3 Nutrient optimization experiments

Nutrient optimization experiments were carried out to determine the lowest concentrations of nitrogen (as ammonium) necessary for bioleaching, and the effect of the addition of phosphorus (as phosphate) on the ability of the enriched cultures to bioleach the secondary ore. A total of nine media containing different concentrations of ammonium and phosphate were used during the experiments (Table 1). Magnesium and ammonium were added as sulfate salts and phosphate and K were added as K_2_HPO_4_, except for media M7 and M8 where K was added as sulfate.

**Table 1:** Media composition used during the experiments and metal concentrations (mg/L), redox potential (mV vs. Ag/AgCl), and dominant genera at the end of the experiments, ± indicates standard deviations for 3 biological samples.

						End values						
		Media composition (mg/L)				Metal concentration (mg/L)						
		Mg^2+^	NH_4_^+^	PO_4_^3−^	K^+^	Co	Cu	Fe	Ni	Zn	Redox potential (mV vs Ag/AgCl)	Dominant genus
30 °C	**M1**	49	874	109	45	2.6±0.0	18.8±0.6	313±4.4	34.2±0.3	91.1±0.7	686±4	*Acidithiobacillus*
	**M2**	49	409	109	45	2.6±0.1	18.9±0.4	305±12	33.8±0.5	89.2±0.5	679±1	*Acidithiobacillus*
	**M3**	49	109	109	45	2.6±0.0	20.2±0.8	306±9.0	34.7±0.4	91.8±1.1	685±1	*Acidithiobacillus*
	**M4**	49	50	109	45	2.5±0.1	20.1±0.5	303±4.3	34.5±0.6	90.8±1.2	686±2	*Acidithiobacillus*
	**M5**	49	25	109	45	2.6±0.0	19.8±0.8	300±3.6	33.9±0.6	90.5±1.0	687±1	*Acidithiobacillus*
	**M6**	49	874	0	45	2.5±0.1	18.3±0.3	260±7.7	34.1±.7	89.8±0.9	679±2	*Acidithiobacillus*
	**M7**	49	0	109	58	2.0±0.0	19.0±0.8	248±2.0	33.1±0.2	83.1±1.6	457±2	*Acidithiobacillus*
	**M8**	49	0	0	58	1.9±0.0	18.0±0.1	230±0.9	32.2±0.5	81.6±1.7	469±5	*Acidithiobacillus*
	**M9**	49	0	0	0	2.0±0.0	16.7±0.4	242±7.5	32.2±0.6	82.6±0.8	482±26	*Acidithiobacillus*
42 °C	**M1**	49	874	109	45	3.0±0.1	23.9±0.3	389±29	37.4±0.4	95.2±0.9	623±6	*Acidithiobacillus*
	**M2**	49	409	109	45	3.0±0.0	23.8±0.6	356±20	37.3±0.5	95.4±1.1	622±5	*Acidithiobacillus*
	**M3**	49	109	109	45	3.0±0.1	24.3±1.0	3234±22	37.3±0.8	96.2±1.5	625±9	*Acidithiobacillus*
	**M4**	49	50	109	45	3.0±0.1	24.1±0.6	331±17	36.6±0.2	94.2±1.7	632±6	*Acidithiobacillus*
	**M5**	49	25	109	45	3.0±0.1	24.4±0.9	356±29	37.1±0.3	95.9±2.3	625±2	*Acidithiobacillus*
	**M6**	49	874	0	45	2.9±0.0	22.9±0.5	330±12	36.5±0.6	93.3±1.6	624±1	*Acidithiobacillus*
	**M7**	49	0	109	58	2.3±0.0	24.8±0.2	535±4.2	36.4±0.7	91.6±2.0	474±8	*Acidithiobacillus*
	**M8**	49	0	0	58	2.3±0.0	26.3±0.6	446±9.2	36.2±0.1	92.7±0.5	475±9	Unknown
	**M9**	49	0	0	0	2.3±0.0	24.2±0.8	496±9.7	35.4±0.3	91.1±1.7	491±8	*Sulfobacillus*
48 °C	**M1**	49	874	109	45	2.9±0.1	25.1±0.6	442±21	36.6±0.5	2.9±1	625±1	*Sulfobacillus*
	**M2**	49	409	109	45	3.0±0.1	26.1±1.6	388±6.9	37.3±0.3	3.0±3	623±4	*Sulfobacillus*
	**M3**	49	109	109	45	2.9±0.2	24.6±1.3	394±24	36.1±1.1	2.9±4	619±4	*Sulfobacillus*
	**M4**	49	50	109	45	3.4±0.2	26.7±1.2	280±15	38.4±0.7	3.4±1	615±5	*Sulfobacillus*
	**M5**	49	25	109	45	3.0±0.0	26.1±0.4	352±24	37.0±0.7	3.0±3	618±2	*Sulfobacillus*
	**M6**	49	874	0	45	2.9±0.2	26.3±0.3	363±15	37.3±0.8	2.9±4	619±2	*Sulfobacillus*
	**M7**	49	0	109	58	2.7±0.1	26.9±1.0	298±9.0	36.8±0.9	2.7±4	532±2	*Sulfobacillus*
	**M8**	49	0	0	58	2.7±0.1	26.6±0.9	349±5.2	36.5±0.6	2.7±3	532±10	*Sulfobacillus*
	**M9**	49	0	0	0	2.6±0.2	26.2±0.9	470±22	36.1±0.7	2.6±2	525±20	*Sulfobacillus*
55 °C	**M1**	49	874	109	45	3.1±0.0	31.9±1.6	161±10	37.6±0.5	95.2±2.3	563±4	Unknown
	**M2**	49	409	109	45	3.3±0.1	32.8±0.9	190±5.2	38.4±0.2	98.5±1.0	575±12	Unknown
	**M3**	49	109	109	45	3.3±0.2	31.3±0.5	213±23	37.7±0.4	96.9±1.3	577±8	Unknown
	**M4**	49	50	109	45	3.1±0.0	32.4±1.2	228±3.7	36.7±0.3	91.7±1.1	589±4	Unknown
	**M5**	49	25	109	45	3.4±0.1	34.2±2.6	204±11	38.5±0.9	98.4±2.0	595±8	Unknown
	**M6**	49	874	0	45	3.1±0.1	32.8±0.8	259±12	38.3±0.2	98.4±0.4	587±4	Unknown
	**M7**	49	0	109	58	2.6±0.1	36.6±8.7	474±240	37.4±0.8	97.5±3.3	477±41	Unknown
	**M8**	49	0	0	58	2.7±0.2	36.0±2.8	386±89	38.3±0.4	97.4±3.4	488±32	Unknown
	**M9**	49	0	0	0	2.8±0.2	34.8±1.4	400±40	38.0±1.0	97.8±3.8	508±22	Unknown
60 °C	**M1**	49	874	109	45	3.1±0.3	37.4±5.9	144±12	38.3±1.8	93.8±6.2	619±4	*Sulfolobus*
	**M2**	49	409	109	45	2.9±0.2	34.2±6.4	164±15	37.4±1.5	92.8±4.5	617±3	*Sulfolobus*
	**M3**	49	109	109	45	2.9±0.1	30.2±0.8	186±15	37.2±0.8	92.2±1.7	612±1	*Sulfolobus*
	**M4**	49	50	109	45	3.0±0.1	26.2±1.9	228±7.9	37.1±0.2	93.1±1.1	623±9	*Sulfolobus*
	**M5**	49	25	109	45	3.0±0.1	30.2±4.9	297±13	37.2±08	93.0±2.7	622±3	*Sulfolobus*
	**M6**	49	874	0	45	3.2±0.1	38.1±6.3	168±43	38.4±1.2	98.1±4.2	625±3	*Sulfolobus*
	**M7**	49	0	109	58	2.3±0.0	36.8±3.6	666±31	37.1±0.5	91.7±0.9	422±5	*Sulfolobus*
	**M8**	49	0	0	58	2.5±0.1	36.6±2.8	463±40	37.8±0.2	94.3±2.7	468±19	*Sulfolobus*
	**M9**	49	0	0	0	2.4±0.1	25.9±4.9	567±30	36.8±0.6	89.8±2.4	437±11	*Sulfolobus*

Cultures were incubated at 30°C, 42°C, 48°C, 55°C, and 60°C in 100 mL of the nine different media (Table 1) and 3% wt./v milled secondary ore. Enrichments were pre-adapted to each media used during the experiments by subsequent subcultures a minimum of four times before inoculation. Triplicate conical flasks were inoculated with 10% v/v of inoculum that contained a mixture of liquid and solids. Cultures were shaken at 150 ppm and grown aerobically for 2 weeks. Redox potential was measured every 2 days to monitor iron oxidation and pH was systematically adjusted to pH 1.8 by the addition of 10% wt./v H_2_SO_4_. Redox potential and pH in the experiments were measured with a HANNA^®^ HI5522 benchtop meter.

Samples for metal (Co, Cu, Fe, Ni, and Zn) analyses were withdrawn at the beginning and at the end of the experiments to assess bioleaching efficiency. Between 10 to 15 mL of sample was passed through a 0.2 μm pore-size sterile filtered and kept at 4°C until further analysis. Samples were diluted with 2% v/v HNO_3_. Metal (Co, Cu, Fe, Ni, and Zn) concentrations in the leachates were analysed using Inductively Coupled Plasma-Optical Emission Spectrometry (ICP-OES) Agilent 5110 VDV at the Camborne School of Mines (Penryn Campus, University of Exeter, United Kingdom).

Biomass for DNA extraction was collected from the inocula and at the end of each of the experiments to determine microbial community composition. Up to 8 mL of a mixture of mineral and liquid from cultures was centrifuged (13,000 rpm for 10 min) and the pellet was washed with TRIS-HCl pH 7.0 buffer to ensure the pH of the pellet was ~7.0 to avoid DNA degradation. The collected pellets with biomass were preserved at −70°C until further processing.

### 3.4 Biomolecular analyses and statistics

DNA was extracted from the pellets using the Qiagen DNeasy PowerLyzer PowerSoil Kit following manufacturer’s instruction. Elution volume was decreased to 50 μL to increase DNA concentration in the extracts. DNA concentration was measured using the Qubit 4.0 Fluorometer dsDNA High Sensibility Assay kit (ThermoFisher Scientific).

Samples were sent to the Centre for Environmental Biotechnology for amplicon sequencing (Bangor University, Bangor, United Kingdom). Libraries of 16 rRNA gene amplicons were prepared as in (Distaso et al., 2020). The hypervariable V4 16S rRNAgene fragment was amplified using the forward F515 (5’-GTGBCAGCMGCCGCGGTAA-3′) and the reverse R806 prokaryotic primers (5’-GGACTACHVGGGTWTCTAAT-3′). The amplified fragments were approximately 290 bp. The primers contained the Illumina adapters and sequencing primers, a 12 bp barcode sequence, a heterogeneity spacer to mitigate the low sequence diversity amplicon issue, and 16S rRNA gene universal primers. PCRs in duplicate were performed using OneTaq Quickload Master Mix (New England Biolabs, United States). All reactions were run with no-template negative controls. Thermocycling conditions were: initial denaturation at 95°C for 2 min, followed by 30 cycles at 95°C for 45 s, 50°C for 60 s, and 72°C for 30 s with a final elongation at 72°C for 5 min. Amplicons were visualized in a 1.5% tris-acetate agarose gels using a GelDoc System (Bio-Rad, CA, United States). DNA bands of approximately 440 bp were gel-purified using NucleoSpin Gel and PCR Clean-up kit (Macherey-Nagel, Germany). The purified amplicons were quantified using Qubit 4.0 Fluorometer (Life Technologies, Carlsbad, CA, United States), pooled in equimolar amounts and the final pool was run on Illumina MiSeq platform (Illumina, San Diego, CA, United States) using 500-cycle v2 chemistry (2 × 250 bp pairedend reads).

Bioinformatic analysis was carried out at the Centre for Environmental Biotechnology (Bangor University, Bangor, United Kingdom). Raw sequencing reads were pre-proceses to extract barcodes from sequences and cleaned of primer sequences using tagcleaner. The barcodes and sequences were rematched using Phyton scripts. The resulting filtered reads were analysed using QIIME v1.3.1. The libraries were demultiplexed based on the different barcodes, followed by classification on operational taxonomic units (OTUs) combining both *de novo* and reference-based methods (openreference OUT generation algorithm) using the SILVA version 138 reference database (Quast et al., 2013).

Data analyses were made in the R environment v 4.1.2 (R Core Team, 2022) and Excel 2019 (Microsoft Corporation, United States). Plots were done in Excel or R 4.1.2 using Rstudio and the *ggplot2* package suite (Wickham, 2016), *MicrobiotaProcess* (Xu et al., 2023), *phyloseq* (McMurdie and Holmes, 2013) and *UpSetR* (Conway et al., 2017) and ComplexUpset v 1.3.5 (Krassowski et al., 2022). OTU abundances were presented as relative abundance in percentage. A matrix of dissimilarities was constructed, using the abundance-based Bray–Curtis dissimilarity index as estimator of beta diversity. The matrix was used to plot samples after Non-Metric Multidimensional Scaling (NMDS) analysis to visualize the similarity of the community composition and Principal Component Analysis (PCoA) to further highlight the taxa that were mostly defining the communities according to the ordination. Upset plots were done to describe how many genera were shared among the different experiments using exclusive intersection among groups, meaning that the elements in an intersection group only belong to that specific intersection group but not to any other set. Further, PERMANOVA on beta diversity allowed us to test the effect of the variables “Medium type” and “Temperature” on the community composition of the samples.

## 4 Results and discussion

### 4.1 Nutrient addition effect on iron oxidation

**Figure 1:**
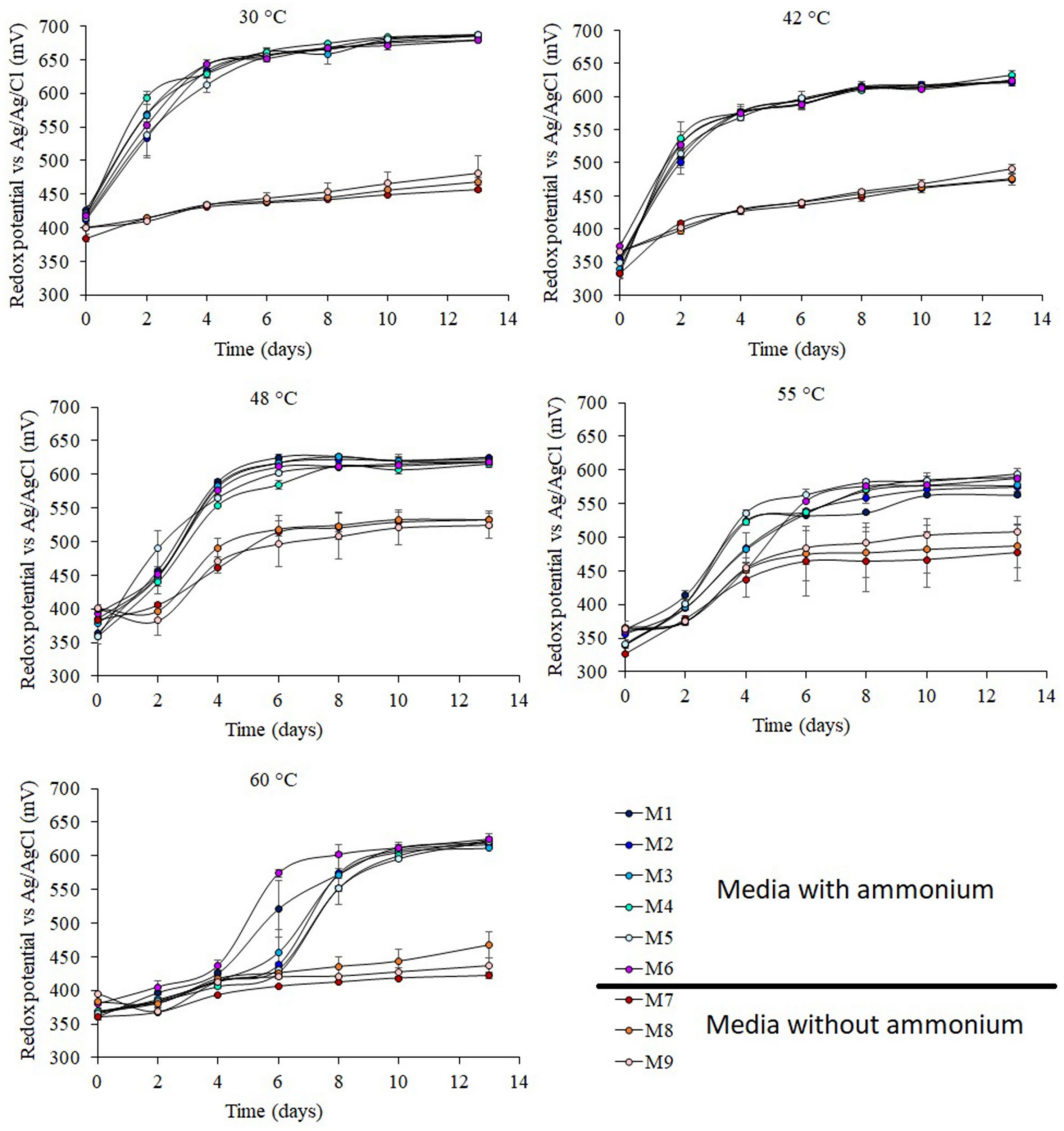
Redox potential of shake flask experiments at 30°C, 42°C, 48°C, 55°C, and 60°C under different concentrations of ammonium and phosphate. Error bars indicate standard deviations for 3 biological samples. Media formulations can be found in Table 1.

Redox potential in acidic environments is directly correlated to Fe speciation (Yue et al., 2016), so it can be used as a proxy for Fe oxidation in acidic environments such as bioleaching. Major differences in redox potential were observed among the different temperatures. The highest redox potential (Figure 1; Table 1) values were achieved at 30°C followed by 42°C, 48°C, and 60°C, with the lowest values obtained at 55°C.

The highest redox potentials were achieved in the experiments carried out in the presence of ammonium (Figure 1; Table 1) independent of the concentration of ammonium used (from 25 mg L^−1^ to 874 mg L^−1^). By contrast, the absence of phosphate did not have any effect on redox potential. In those experiments that were not amended with ammonium, the redox potential was similar to the control experiments (medium M9) that did not contain ammonium, phosphate or K. Experiments conducted at 30°C showed a redox potential of 684 ± 3 mV (*vs* Ag/AgCl) in the presence of ammonium while those experiment without ammonium the redox potential was 469 ± 12 mV (*vs* Ag/AgCl). The redox potential at 42°C had a similar profile to that of the redox potential at 30°C, with a short lag phase and higher redox potential in experiments with ammonium (625 ± 4 mV *vs* Ag/AgCl) than those without ammonium (480 ± 9 mV *vs* Ag/AgCl). At 48°C, the experiments without ammonium had a 2-day lag phase followed by a small increase of redox potential to 530 ± 4 mV (*vs* Ag/AgCl). In the experiments with ammonium the redox potential achieved was only 90 mV higher than cultures grown in the absence of ammonium (Figure 1). The lowest redox potentials in experiments with ammonium were obtained at 55°C (581 ± 12 mV *vs* Ag/AgCl), and again, in the experiments without ammonium the redox potential was 90 mV lower. The redox potentials at 60°C had a lag phase of 5–6 days in the experiments with ammonium, but after that, the redox potential increased to 620 ± 5 mV (*vs* Ag/AgCl). The experiments without ammonium showed a small increase in redox potential between the start and end of the experiments of just 42–85 mV (*vs* Ag/AgCl), remaining at 442 ± 24 V (Ag/AgCl) at the end of the cultivation period.

These results are similar to those of Hubau et al. (2024) and Falagán et al. (2024) who showed that ammonium was the only nutrient that affected the microbial iron oxidation during the bioleaching of the Sotkamo secondary ore. The dissolution of the mineral may provide some of these nutrients such as phosphate and K. Phosphate and K are commonly found in rocks and ores that dissolved during bioleaching and would be available for the microorganism uptake. For example, the dissolution of apatite (Ca_5_(PO_4_)_3_(OH,F,Cl)) could be a source of phosphate while the dissolution of minerals such as muscovite (KAl_2_(AlSi_3_O_10_)(F,OH)_2_) could provide K.

### 4.2 Nutrient addition effect on metal dissolution

The amounts of Co, Cu, Ni and Zn dissolved were lowest at 30°C, whereas the dissolved metal concentrations at 42°C, 48°C, 55°C, and 60°C metal dissolution were similar, except for Cu that was higher at 55°C and at 60°C.

The effect of nutrient addition on metal dissolution varied depending on the temperature at which the experiment was carried. At 30°C, Ni dissolution was lower in the experiments without ammonium while at the other temperatures was similar in experiments with and without ammonium. The similar dissolution yields of Ni in all the experiments are likely caused by the nature of the secondary ore. This is due to the presence of soluble sulfate salts with high nickel content that dissolved rapidly when in contact with acidic solutions (Falagán et al., 2024).

At 30°C, Zn dissolution was 9% lower in experiments without ammonium than with ammonium (Table 1), but in the experiments at higher temperatures, Zn dissolution was similar in all the experiments independently of nutrient addition. Sphalerite is the main Zn-bearing mineral in the secondary ore (Hubau et al., 2020) and, although this mineral can be dissolved by acid only, its bioleaching is enhanced due to Fe oxidation (Fowler and Crundwell, 1998; Schippers et al., 2019; Abdollahi et al., 2022). Different nutrient requirements by the dominant microbial species at 30°C than the other species at the higher temperatures (42°C, 48°C, 55°C, and 60°C) may have caused lower microbial Fe-oxidation activity and therefore cause lower Fe(III) availability for the oxidation of sphalerite.

**Figure 2:**
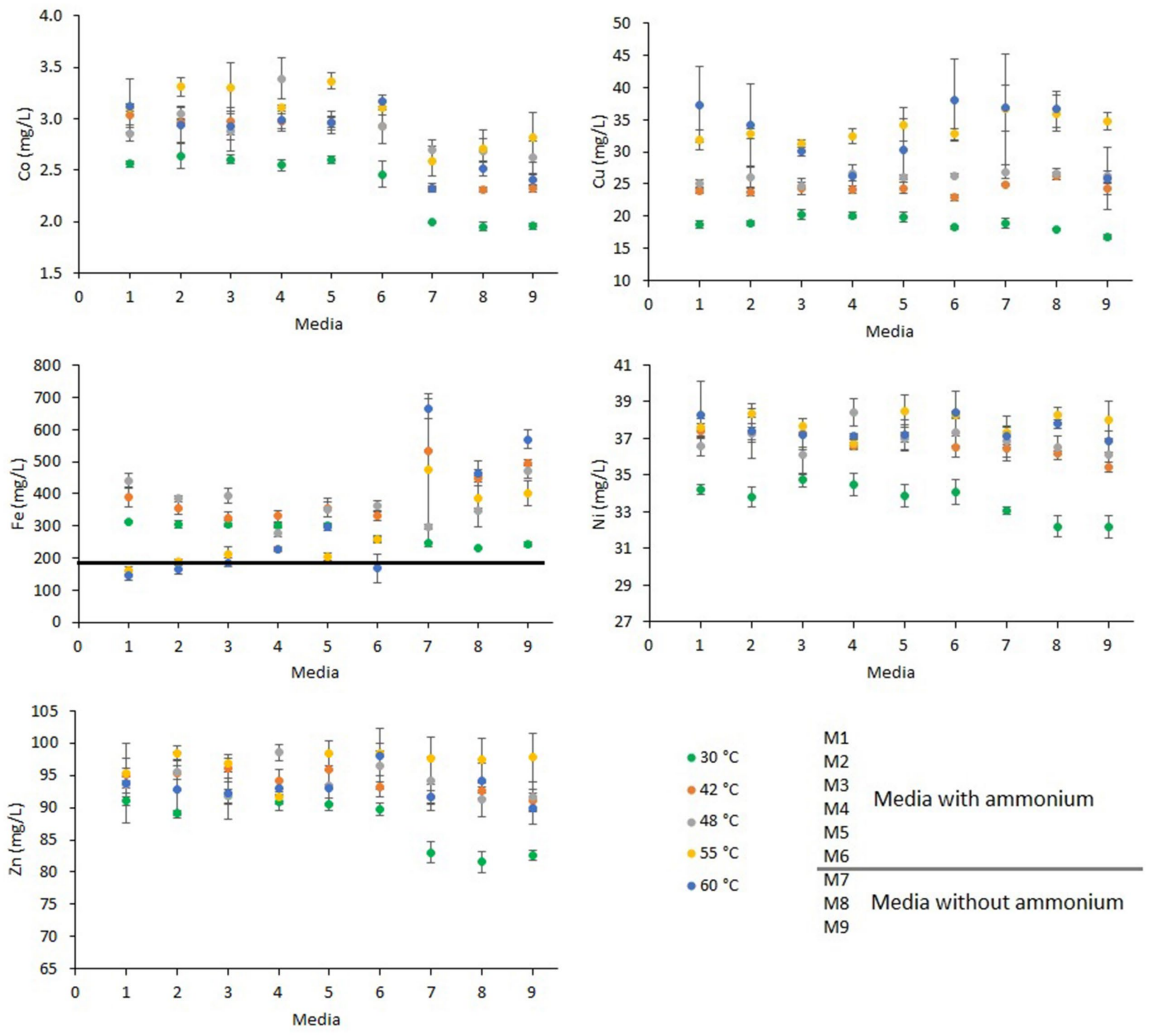
Metal (Co, Cu, Fe, Ni, and Zn) concentrations (mg/L) at the end of the shake flask experiments at 30°C, 42°C, 48°C, 55°C, and 60°C under different concentrations of ammonium and phosphate. The line on the Fe graph represents metal concentration at the beginning of the experiments. For other metals (Co, Cu, Ni, and Zn), the metal dissolutions at the beginning of the experiments was below the lowest values depicted on the axis. Error bars indicate standard deviations for 3 biological samples. Media formulations can be found in Table 1.

Cobalt dissolution was 11–24% lower in those experiments without ammonium than in the experiments with ammonium (Figure 2; Table 1). This metal was mainly contained in the pyrite (Hubau et al., 2020), which is a bioleachable mineral (Yahya and Johnson, 2002). The depletion of ammonium caused lower microbial Fe-oxidizing activity which is reflected by the low redox values of experiments where ammonium was absent (Figure 1). Low ammonium concentration (101 mg/L ammonium) also caused lower Co dissolution during the bioleaching of secondary ore in batch reactors with higher concentration of ammonium (1,009 mg/L ammonium) (Hubau et al., 2024). This highlights the necessity of adding ammonium to promote microbial Fe-oxidation during bioleaching.

Copper dissolution was mostly temperature dependent as the lowest dissolution amounts were found at 30°C, followed by 42°C and 48°C, with the highest Cu dissolution at 55°C and 60°C. Copper is mainly present in chalcopyrite in the secondary ore (Hubau et al., 2020) whose leaching is improved at high temperatures (Rodrıguez et al., 2003; Petersen and Dixon, 2006; Córdoba et al., 2008; Watling, 2014).

Iron dissolution varied greatly compared to the other metals. At 30°C, 42°C, and 48°C, Fe decreased as the ammonium concentration in media decreased, opposite to the trend recorded for the 55°C and 60°C experiments. At 30°C, Fe in solution without ammonium was similar in the three experiments. At the other temperatures in media without ammonium and phosphate the leachates had higher concentrations of Fe than in experiments with these two nutrients. The different iron dissolutions may have been caused by pH variations during the experiments. As pH was adjusted every 2 days to 1.8, the pH reached values over 2.0, mostly at the beginning of the experiments, likely causing the precipitation of iron as Fe(III) minerals (e.g., jarosite). Precipitation of jarosite is favored at pH >2.0, the combination of high pH, high temperature, and ammonium and K may have contributed to the formation of jarosites including ammoniojarosite (Tuovinen and Carlson, 1979; Lazaroff et al., 1982; Wang et al., 2007; Kaksonen et al., 2014; Sandy Jones et al., 2014). Removing nutrients and K from the solution may have favored the retention of Fe (Figure 2). Further studies should be carried out where pH is controlled to determine iron dissolution and precipitations rates at different nutrient concentrations.

### 4.3 Microbial community composition

Some of the collected samples did not yield enough DNA to perform amplicon sequencing; these were: at 30°C, M4 start; at 42°C, M8 end and M9 start; at 48°C, M2 start, M6 start, M8 start and M9 start; at 55°C, all of the samples; at 60°C, M7 start and M8 start.

**Figure 3:**
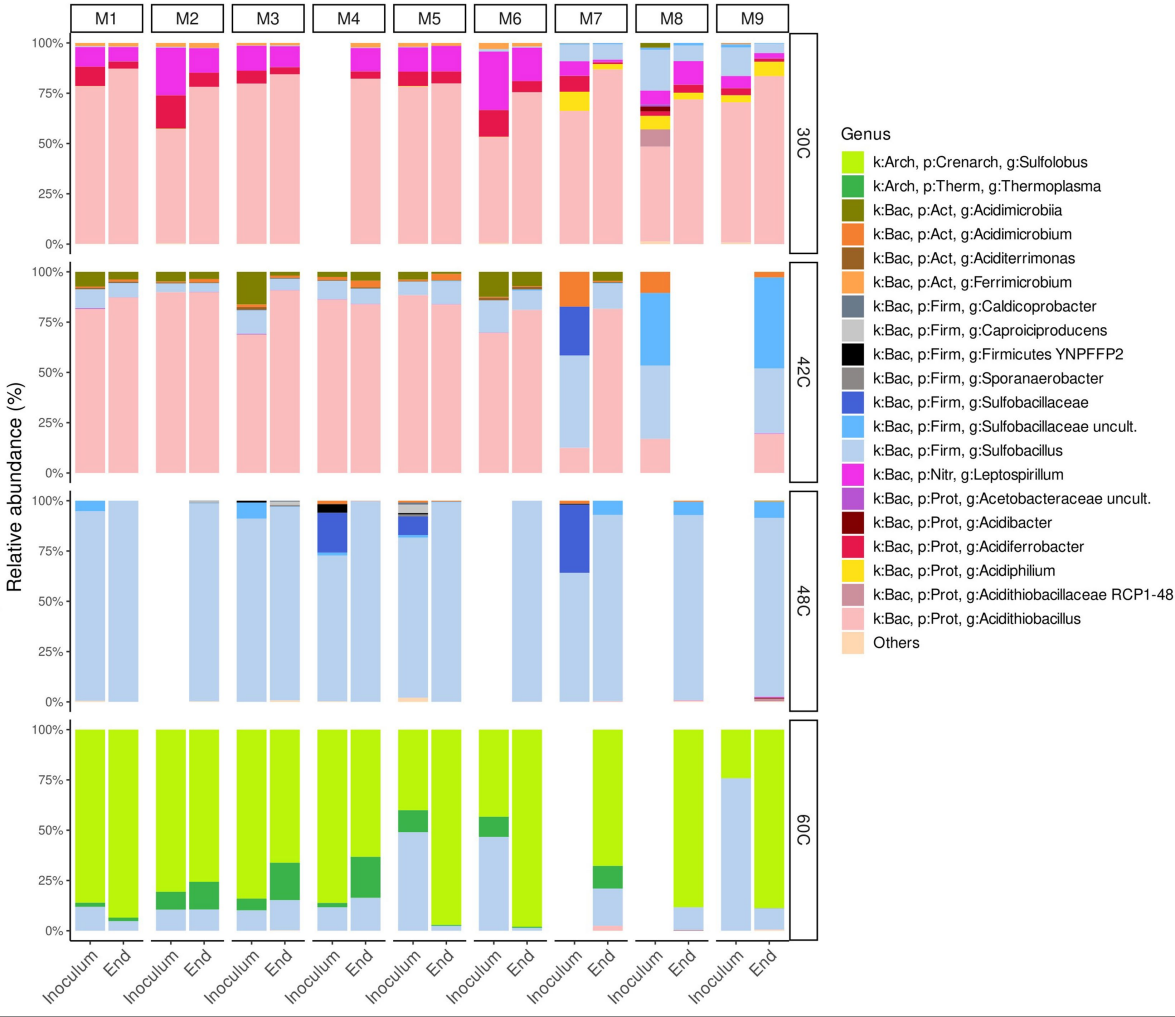
Relative abundance at genus level of microbial communities in the inocula and at the end of the experiments at 30°C, 42°C, 48°C, and 60°C under different concentrations of ammonium and phosphate. Blank spaces correspond to samples that did not yield any DNA. Media formulations can be found in Table 1.

At 30°C, the microbial community at the end of all the experiments was dominated by *Acidithiobacillus* sp. (72–87%) with other species at lower relative abundance belonging to the genera *Leptospirillum* sp., *Sulfobacillus* sp., *Acidiferrobacter* sp., and *Ferrobacter* sp. (Figure 3). In media that contained ammonium (M1 to M7), the second dominant microorganisms were genera *Leptospirillum* sp. and *Acidiferrobacter* sp., whereas in media that did not contain ammonium the second dominant organisms were *Leptospirillum* sp. and *Sulfobacillus* sp. with reduced relative abundances of *Acidiferrobacter* sp. and the appearance of the heterotroph *Acidiphilium* sp. (Figure 3).

**Figure 4:**
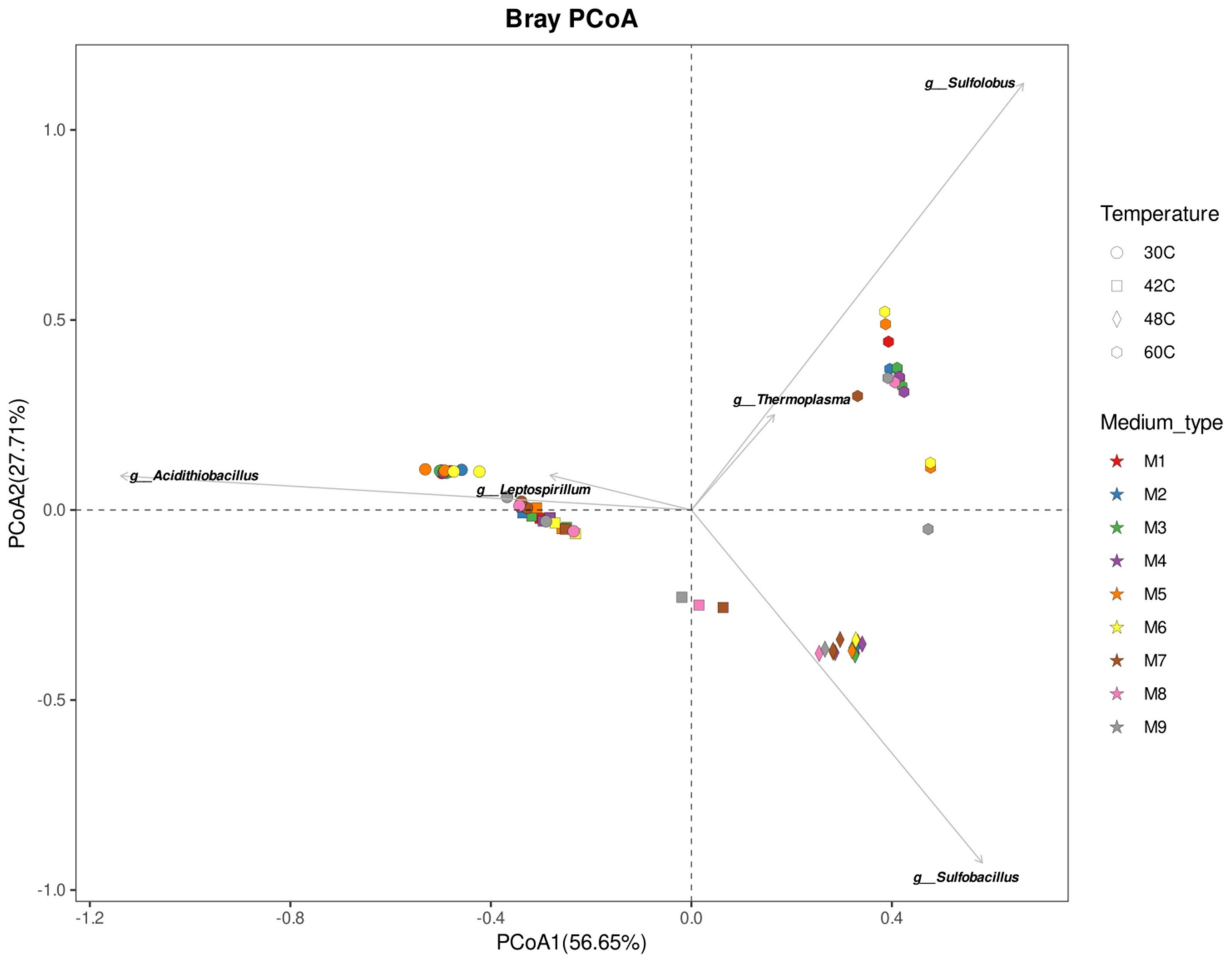
Principal Component Analysis ordination illustrates the compositional similarity of microbial communities at the end of the experiments at 30°C, 42°C, 48°C, and 60°C under different concentrations of ammonium and phosphate. The top five genera driving groups ordination are plotted on top of the PCoA. Media formulations can be found in Table 1.

At 42°C, the microbial communities at the end of the experiments with media containing ammonium (M1 to M7) were dominated by *Acidithiobacillus* sp. (82–91%), with lesser amounts of species at belonging to the genera *Sulfobacillus* sp. and *Acidimicrobium* sp. (Figures 3, 4). The microbial community in the experiments without ammonium (M7 to M9 media) shifted towards a composition closer to that found at 48°C (Figure 4). For example, at the end of the experiment with medium M9 the microbial community was dominated by *Sulfobacillus* sp. (77%), with lesser amounts of *Acidithiobacillus* sp. (19%) (Figure 3). Although, the end of experiment with medium M8 did not yield enough DNA, the end of experiment with M9 suggest that nutrient depletion causes a shift of microbial community towards a *Sulfobacillus*. This is particularly interesting as the sulfobacilli are spore-forming bacteria (Norris et al., 1996), spores allow survival after being exposed to unfavorable environmental conditions, whereas the acidithioballi do not form spores, making their survival under unfavorable environmental conditions limited and ultimately impossible. In addition, *Sulfobacillus* spp. are mixotrophs that may be using the organic carbon released when cells lyse outcompeting other microorganisms at low concentrations of nutrients and, when favourable conditions return, *Sulfobacillus* spp. proliferates over other species.

The microbial community at 48°C was dominated by *Sulfobacillus* sp. all the samples either the inoculum or at the end of the experiments with relative abundances of 90–100% (Figure 3) with very low relative abundances of other genera.

At 60°C, the microbial communities at the end of all the experiments were dominated by *Sulfolobus* sp. (63–98%) with lower relative abundances of *Sulfobacillus* sp. (1–19%) and *Thermoplasma* sp. (0–20%) (Figure 3).

**Figure 5:**
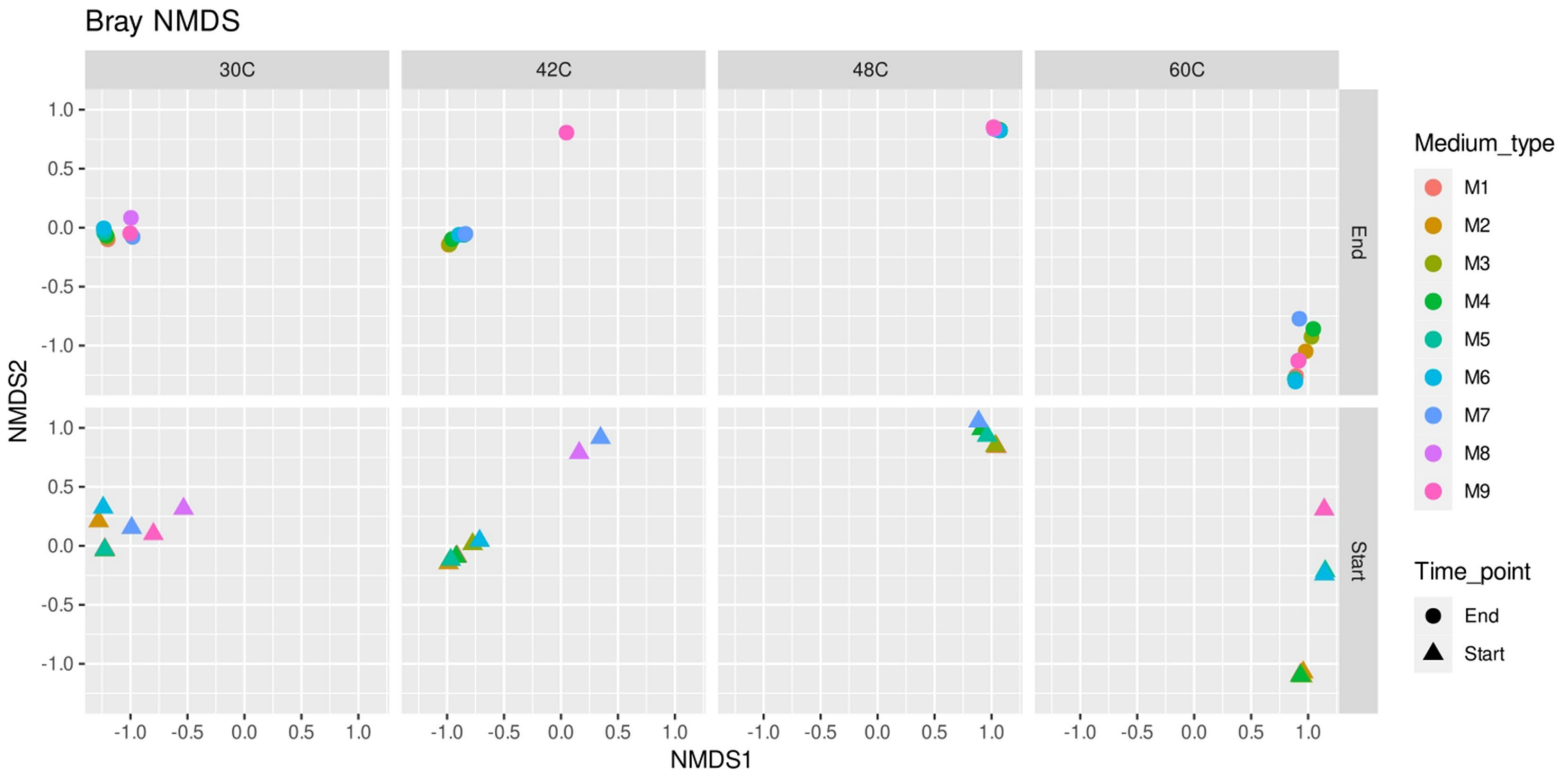
Non-metric multidimensional scaling (NMDS) analysis plot of microbial communities in inocula (start) and at the end of the experiments at 30°C, 42°C, 48°C, and 60°C under different concentrations of ammonium and phosphate. Media formulations can be found in Table 1.

**Figure 6:**
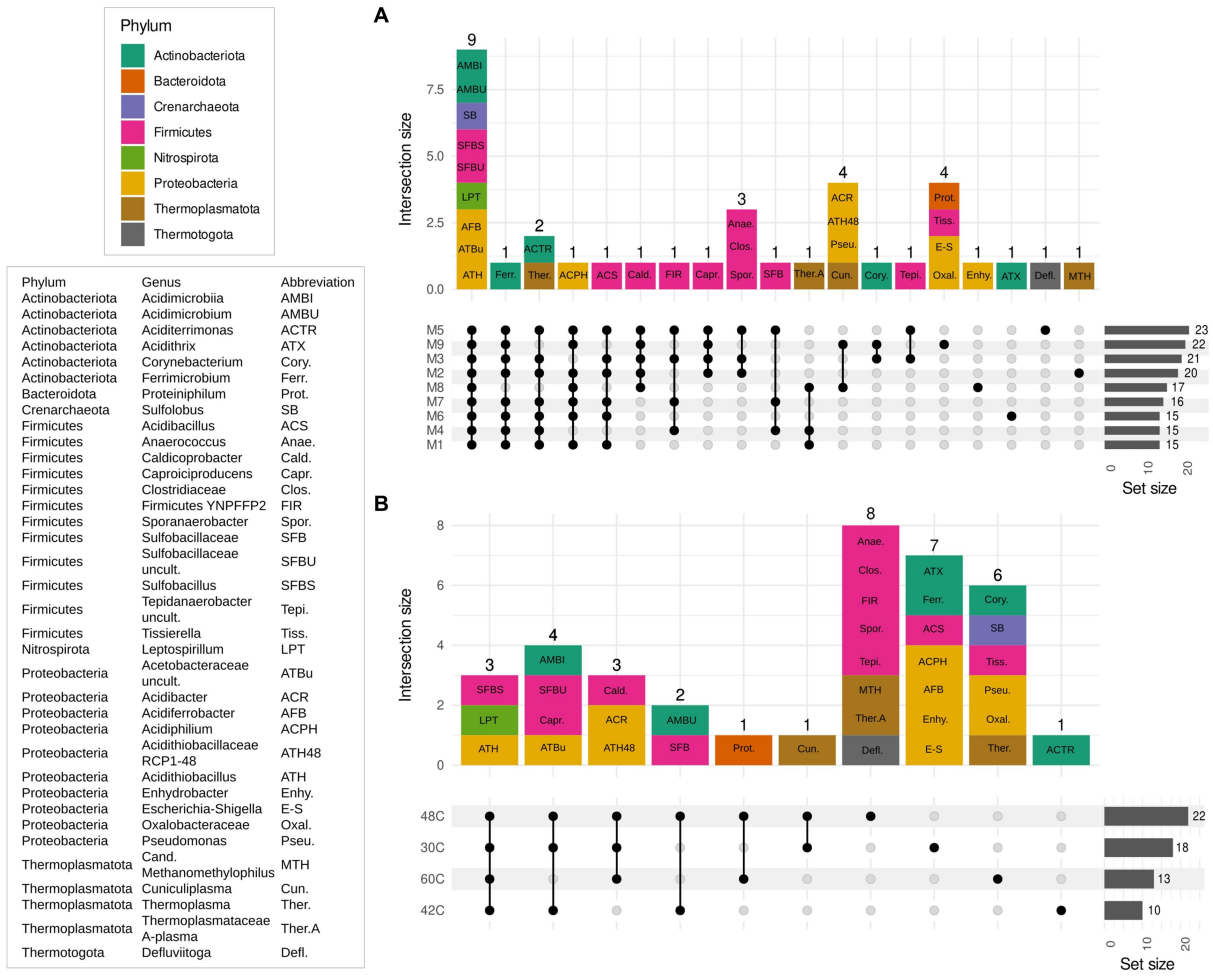
Upset plots show intersecting sets of samples (exclusive intersection), regarding medium composition **(A)** and temperature **(B)**. The set size indicates the number of genera present for each experiment and the intersection size indicates the number of genera that are shared among the members of an intersection group. Abbreviations for the genera shared by each group are indicated in each count unit and its phylum is color coded. Key for the abbreviations used in the intersection bar chart are explained in the box on the left. Media formulations can be found in Table 1.

Microbial communities were similar at the beginning and at the end of the experiments, which is expected as the enrichments were adapted to each medium previously to the start of the experiments (Figures 3, 5). The main factor that shaped the microbial communities was the temperature (PERMANOVA: *R*^2^ = 0.84, *p*. value = 0.0001; statistical results for the PERMANOVA can be found in Supplementary Table S1) while the medium type showed a very weak yet significant effect on the community composition (*R*^2^ = 0.04, *p*. value = 0.007); (Figures 3–5), media without ammonium and phosphate selected for some genera (M9 medium selected for 5 genera and M8 and M9 selected for further 4 genera, Figure 6). Nine genera were the most widely distributed genera being present in all experiments: *Acidimicrobiia* (class), *Acidimicrobium*, *Sulfolobus*, *Sulfobacillus*, an uncultured *Sulfobacillaceae* (Family), *Leptospirillum*, *Acidiferrobacter*, uncultured *Acetobacteraceae* (Family) and *Acidithiobacillus* (Figure 6). Other genera were present only present in some of the experiments, for instance three Firmicutes, *Anaerococcus*, *Clostridiaceae* (Family) and *Sporanaerobacter*, were present in experiments with media M5, M2 and M3 while three Proteobacteria and the archaeon *Cuniculiplasma* were present in experiments with media M9 and M8. Experiments with media M9 showed the highest number of exclusive genera. Experiments carried out at 48°C and 30°C showed the highest number of genera (respectively 22 and 18). The temperature factor showed higher exclusive genera and lower number of shared genera when compared to the different media used. Only three genera are present in all the temperatures: *Sulfobacillus, Leptospirillum* and *Acidithiobacillus.* The experiments carried out at 48°C showed the highest number of exclusive genera (*n* = 8), followed by 30°C (*n* = 7) and 60°C (*n* = 6), while the 42°C treatment has one exclusive genera: *Aciditerrimonas* (Figure 6).

As the different microorganisms found at the end of the experiments (e.g., *Acidithiobacillus, Sulfobacillus,* and *Sulfolobus*) have different optimal temperatures for growth, it was expected that temperature was the dominant variable shaping the microbial communities. The optimal temperatures for growth of the mesophilic and moderate thermophiles of the genus *Acidithiobacillus* sp. are ~30°C and 45°C, respectively (Mirete et al., 2017); *Sulfobacillus* sp. are moderate thermophiles with optimum growth temperatures of 38–55°C (Norris et al., 1996; Melamud et al., 2003; Bogdanova et al., 2006; Johnson et al., 2008; Zhang et al., 2021). *Acidithiobacillus* spp. and *Sulfobacillus* spp. are iron and sulfur oxidizing bacteria (Dopson, 2016) suggesting both groups were driving iron oxidation during the experiments at temperatures between 30°C and 55°C. At 60°C, the dominant genus was *Sulfolobus* sp. (including species now belonging to the genus *Sulfuracidifex*; Itoh et al., 2020) which are thermophilic archaea with optimum growth temperatures of 65–80°C (Brock et al., 1972; Huber and Stetter, 1991; Suzuki et al., 2002; Itoh et al., 2020). Some of these archaea are able to oxidize iron and sulfur (Golyshina et al., 2016; Itoh et al., 2020), the high redox potentials at 60°C suggest that the microbial community was dominated by those species able to oxidize iron.

## 5 Conclusions and further research

The low nutrient concentrations used for the bioleaching of the secondary ore did not have a noticeable effect on the microbial communities, exception made for the treatment at 42°C whose irrigation solutions did not contain phosphate and ammonium. Responses of microorganisms used in bioleaching under low nutrient concentrations have not been studied either at the species level or at community level. Nutrient availability and intake rates may be factors that would cause microbial species shifts during bioleaching thus causing the dominance or disappearance of certain species. This needs further investigation as there is a lack of existing studies addressing this matter.

The dominance of *Sulfobacillus* spp. under nutrient depletion is particularly interesting, the mixotrophic nature of this genus combined with the spore-forming capacity may favor the proliferation of *Sulfobacillus* spp. over autotrophs and other non-spore forming species. Further experiments to determine the effect of nutrients on microbial communities should be considered when mixotrophic spore-forming (e.g., sulfobacilli) microorganisms and non-mixotrophs (e.g., acidithiobacilli) microbes coexist at 42°C, a temperature equal or close for their optimal growth.

There are other parameters that should be considered when performing nutrient optimization such as pulp density. The study presented here was performed at a relatively low pulp density (3% wt./v) and in small volumes (100 mL) with a secondary ore that contained a high abundance of gangue minerals. Further experiments on nutrient optimization should be performed to understand microbial nutrient consumption at different pulp densities. Presumably, higher pulp densities would require increasing nutrient concentrations to support the growth of the microbial community during batch experiments.

However, during bioleaching in continuous mode such as laboratory-scale columns, nutrients are added continuously, thus lower concentration of nutrients may be sufficient. Monitoring nutrient consumption is recommended in order to understand the nutrient requirements of the microbial communities during the different phases of growth during bioleaching. This will help to ascertain whether the addition of nutrients is necessary during the full period of the bioleaching process. In laboratory-scale columns or heap bioleaching, for example, addition would only be necessary during the microbial exponential growth phase.

Microbial nutrient requirements in bioleaching environments should be studied further to understand how this affect Fe oxidations rates which in turn would influence metal dissolution rates. The present study demonstrates that addition of ammonium during the bioleaching of the secondary Sotkamo ore using indigenous microorganisms is a requirement for improving Fe oxidation and enhancing metal extraction rates, while the addition of other nutrients (K and phosphate) does not provide any advantages towards higher bioleaching performance. The addition of nutrients such as ammonium may favor the precipitation of secondary minerals such as jarosite. The optimization of nutrient addition may prevent the formation of undesirable minerals that may hinder metal extraction rates through removal of Fe(III), co-precipitation or passivation of mineral particles.

In conclusion, a nutrient medium with low concentration of ammonium is recommended for the bioleaching of the secondary ore and other low-grade ores and wastes, deeming unnecessary the addition of phosphate, K, and other micro- and macronutrients. However, the concentration of ammonium should be optimized for the bioleaching conditions of the different ores in order to optimize microbial growth and metal extraction rates.

## Data availability statement

The sequence data generated in this study have been deposited in the NCBI Sequence Read Archive (SRA; https://www.ncbi.nlm.nih.gov/sra/?term=PRJNA1081817) under Bioproject PRJNA1081817 (https://www.ncbi.nlm.nih.gov/bioproject/PRJNA1081817). The public database SILVA non-redundant SSU Ref database, https://www.arb-silva.de/, v.138, was used in this study.

## Author contributions

CF: Conceptualization, Formal analysis, Investigation, Methodology, Writing – original draft, Writing – review & editing. TS: Formal analysis, Writing – review & editing, Data curation, Methodology. GW: Methodology, Writing – review & editing. RB: Writing – review & editing, Data curation, Formal analysis, Methodology. DD: Writing – review & editing, Conceptualization. KH-E: Project administration, Writing – review & editing.
