# Supplementary material for: Nutrient optimization in bioleaching: are we overdosing?
Source: Front Microbiol. 2024 May 17;15:1359991. doi: 10.3389/fmicb.2024.1359991 (PMC11140130; doi:10.3389/fmicb.2024.1359991)
Supplement: Supplementary file 1 [file Table_1.DOCX]

**Nutrient optimization in bioleaching: are we overdosing?**

# Carmen Falagan^1^*^¥^, Tomasa Sbaffi^2,3^, Gwion B. Williams^4^, Rafael Bargiela^4^, David W. Dew^1^, Karen A. HudsonEdwards^1^

^1^Environment & Sustainability Institute and Camborne School of Mines, University of Exeter, Penryn, Cornwall, TR10 9FE, UK

^2^Molecular Ecology Group, Water Research Institute (IRSA), National Research Council of Italy (CNR), Largo Tonolli 50, 28922 Verbania, Italy

^3^National Biodiversity Future Center, NBFC, Piazza Marina 61, 90133 Palermo, Italy

^4^Centre for Environmental Biotechnology (CEB), School of Natural Sciences, Thoday Building, 2nd floor, Deiniol Road, Bangor University, Bangor, Gwynedd, LL57 2UW, UK

***Correspondence:** carmen.falagan@port.ac.uk

^¥^Current address: School of Biological Sciences, King Henry Building, King Henry 1^st^ St.; University of Portsmouth; Portsmouth; PO1 2DY; United Kingdom.

**Supplementary File**

**Supplementary Table 1.** Statistical results for the PERMANOVA, assessing the influence of temperature and medium type on the community composition.

|  | **Df** | **Sums Sq** | **Mean Sq** | **F-Model** | **R^2^** | **p-value** |
| --- | --- | --- | --- | --- | --- | --- |
| Medium_type | 8 | 0.57 | 0.07 | 2.11 | 0.04 | 0.0072 |
| Temperature | 3 | 12.52 | 4.17 | 123.70 | 0.85 | 0.0001 |
